# Traumatic tricuspid insufficiency with a ventricular septal defect in a child: a case report

**DOI:** 10.11604/pamj.2019.33.265.19747

**Published:** 2019-07-29

**Authors:** Hicham El Malki, Hanae Bouhdadi, Aziz Belkhadir, Jaafar Rhissassi

**Affiliations:** 1 Pediatric Cardiovascular Surgery Unit, Children's Hospital, Rabat, Morocco

**Keywords:** Blunt chest trauma, tricuspid insufficiency, ventricular septal defect

## Abstract

Traumatic tricuspid insufficiency is very rare. In this report we describe an interesting case of a 13-year-old boy who suffered chest trauma from a horse kick. Echocardiography demonstrated a remarkable tricuspid regurgitation with ventricular septal defect. Once assessing the diagnosis, an emergency open heart surgery was necessary to repair the injuries with good results.

## Introduction

Blunt chest trauma leads to a wide range of lesions, relatively minor parietal injuries to potentially fatal cardiac lesions, making diagnosis and management difficult. The tricuspid valve is rarely involved [1], the interventricular septum defect is mostly encountered during penetrating trauma. We report a case of severe tricuspid insufficiency associated with an apical muscular Ventricular Septal Defect (VSD) in a thirteen year old patient following a horse kick. Management strategies are discussed and our treatment approach is presented.

## Patient and observation

**Figure 1:**
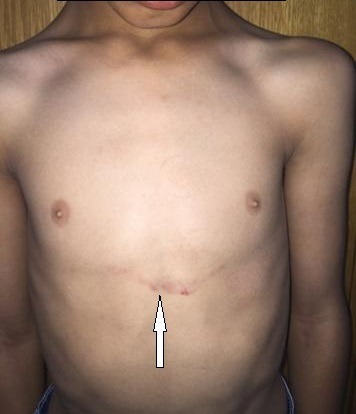
Ecchymotic lesion regarding the epigastric area

**Figure 2:**
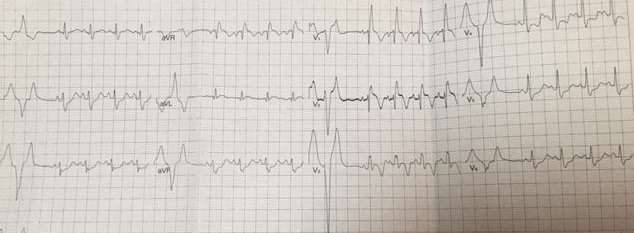
A complete right bundle branch block and polymorphic ventricular extrasystoles

**Figure 3:**
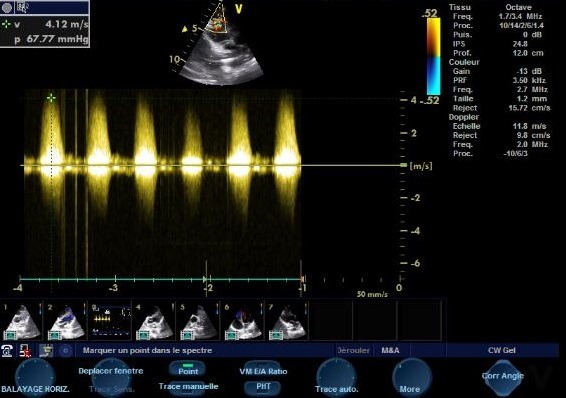
TTE: apical muscular VSD

**Figure 4:**
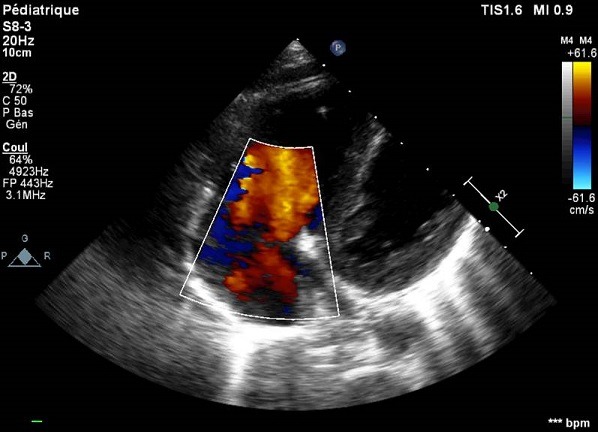
TTE: severe tricuspid regurgitation

**Figure 5:**
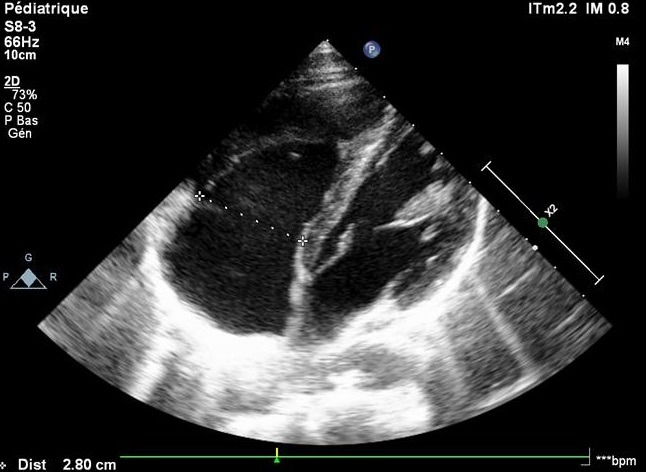
TTE: dilated right atrium

**Figure 6:**
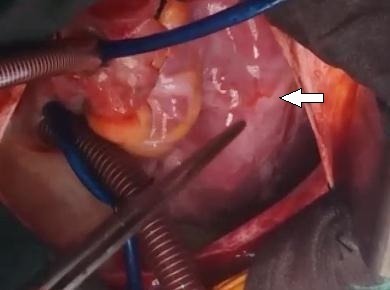
Surgical view: a myocardial contusion

**Figure 7:**
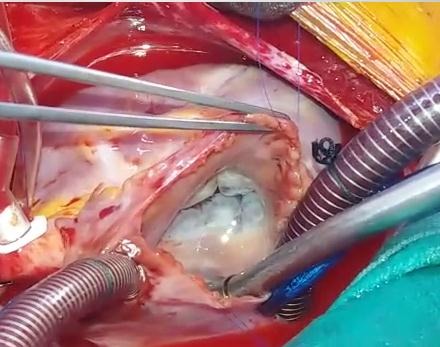
Surgical view: tricuspid repair with devega annuloplasty

A 13-year-old, living in a rural area, with no cardiovascular disease history, was admitted to the pediatric emergency department for dyspnea, with the notion of thoracic trauma by a horse kick six days earlier. The physical examination finds an orthopneic patient with signs of right heart failure and an ecchymotic lesion regarding the epigastric area (Figure 1). Cardiac auscultation revealed holosystolic murmur. The electrocardiogram showed a complete right bundle branch block and polymorphic ventricular extrasystoles (Figure 2). The chest X-ray was unremarkable. Transthoracic echocardiography revealed an apical muscular VSD associated to severe tricuspid regurgitation, a dilated right atrium, and normal left ventricular function (Figure 3, Figure 4, Figure 5). The child was referred for surgery. He underwent, through a median sternotomy, an open heart surgery using routine cardiopulmonary bypass between the ascending aorta and the two laced vena cava veins. There was a small pericardial effusion and a myocardial contusion (Figure 6). After aortic cross-clamping and myocardial protection, the right atrium was opened. Tricuspid valve repair was performed with reimplantation of the ruptured papillary muscle and a devega annuloplasty (Figure 7). The complete closure of the interventricular septal defect could not be achieved because of its situation which rendered it inaccessible. The immediate postoperative period was uneventful with a short stay (48 hours) in the intensive care unit (ICU). At the 6 month follow up echocardiography showed satisfactory leaflet coaptation without tricuspid regurgitation and persistence of a small left to right shunt which will be re-evaluated.

## Discussion

Valvular lesions following blunt thoracic injury are uncommon. Tricuspid insufficiency is thought to be a rare complication of blunt, non-penetrating chest trauma. The incidence of blunt chest wall trauma and reported traumatic tricuspid regurgitation has been increasing during the last decade [1]. However, the diagnosis is difficult because this pathology slowly progress and its presentation can be atypical or asymptomatic, so its incidence rates may be underestimated. The most common mechanism of acute or subacute tricuspid regurgitation is an anteroposterior compression of the chest with a sudden increase in the right ventricular pressure during the end diastolic phase, when the main pulmonary vessels are compressed. This generates a marked traction on both valvu¬lar and subvalvular apparatus. VSD is a particularly uncommon result [2, 3]. Its severity, presentation and course are variable, with presenting signs often masked by concomitant injuries, [4] and the presentation of the murmur is often delayed. The pathogenesis of VSD following blunt chest trauma is unclear but is thought to be caused by either early mechanical rupture or delayed inflammatory rupture.

Mechanical septal rupture has been proposed to occur as the heart is compressed during late diastole, after atrial contraction, when the ventricles are filled and the valves closed. This may occur as a result of direct cardiac impact or when the heart is compressed between the sternum and the spine [5]. It has also been suggested that a healed congenital VSD with a weakened ventricular septum may re-open with significant blunt trauma to the chest [4]. Delayed inflammatory rupture is thought to occur when cardiac injury causes localised edema with disruption of microvascular flow, leading to infarction, septal liquefaction, and perforation. Small asymptomatic traumatic VSDs may be managed conservatively as they often close spontaneously [6]. Surgical repair is indicated if the defect is large, if the pulmonary to systemic blood flow ratio exceeds 2/1, or if there is evidence of cardiac failure [5]. However, a persisting small lesion with chronic left to right shunting may with time result in right ventricular failure. Cases of blunt chest trauma resulting in a combined lesion of traumatic VSD and tricuspid insufficiency, as in our patient, were also reported by others [7]. Signs of heart failure with elevated pulmonary pressure and a significant left-to-right shunt dominated the clinical picture in most patients.

## Conclusion

This case reminds us that physicians in the emergency department should be aware of this potential complication following non-penetrating chest trauma. Early operation should be emphasized to achieve good functional results and preserve the right ventricular function.

## Competing interests

The authors declare no competing interests.
